# 10-Year Prospective Clinical and Radiological Evaluation After Matrix-Induced Autologous Chondrocyte Implantation and Comparison of Tibiofemoral and Patellofemoral Graft Outcomes

**DOI:** 10.1177/03635465241227969

**Published:** 2024-02-21

**Authors:** Jay R. Ebert, Minghao Zheng, Michael Fallon, David J. Wood, Gregory C. Janes

**Affiliations:** † School of Human Sciences (Exercise and Sport Science), University of Western Australia, Crawley, Perth, Western Australia, Australia; ‡ HFRC Rehabilitation Clinic, Nedlands, Perth, Western Australia, Australia; § School of Surgery (Orthopaedics), University of Western Australia, Crawley, Perth, Western Australia, Australia; ‖ Perth Radiological Clinic, Subiaco, Perth, Western Australia, Australia; ¶ Perth Orthopaedic and Sports Medicine Centre, West Perth, Perth, Western Australia, Australia; Investigation performed at University of Western Australia, Crawley, Perth, Western Australia, Australia

**Keywords:** matrix-induced autologous chondrocyte implantation, clinical outcomes, magnetic resonance imaging

## Abstract

**Background:**

Long-term outcomes in larger cohorts after matrix-induced autologous chondrocyte implantation (MACI) are required. Furthermore, little is known about the longer-term clinical and radiological outcomes of MACI performed in the tibiofemoral versus patellofemoral knee joint.

**Purpose:**

To present the 10-year clinical and radiological outcomes in patients after MACI and compare outcomes in patients undergoing tibiofemoral versus patellofemoral MACI.

**Study Design:**

Case series; Level of evidence, 4.

**Methods:**

Between September 2002 and December 2012, 204 patients who underwent MACI were prospectively registered into a research program and assessed preoperatively and at 2, 5, and 10 years postoperatively. Of these patients, 168 were available for clinical review at 10 years, with 151 (of a total of 182) grafts also assessed via magnetic resonance imaging (MRI). Patients were evaluated using the Knee injury and Osteoarthritis Outcome Score, a visual analog scale for pain frequency and severity, satisfaction, and peak isokinetic knee extensor and flexor strength. Limb symmetry indices (LSIs) were calculated for strength measures. Grafts were scored on MRI scans via the MOCART (magnetic resonance observation of cartilage repair tissue) system, with a focus on tissue infill and an overall MRI graft composite score.

**Results:**

All patient-reported outcome measures improved (*P* < .0001) up to 2 years after surgery. Apart from the significant increase (*P* = .004) in the peak isokinetic knee extensor LSI, no other patient-reported outcome measure or clinical score had changed significantly from 2 to 10 years. At the final follow-up, 92% of patients were satisfied with MACI to provide knee pain relief, with 76% satisfied with their ability to participate in sports. From 2 to 10 years, no significant change was seen for any MRI-based MOCART variable nor the overall MRI composite score. Of the 151 grafts reviewed via MRI at 10 years, 14 (9.3%) had failed, defined by graft delamination or no graft tissue on MRI scan. Furthermore, of the 36 patients (of the prospectively recruited 204) who were not available for longer-term review, 7 had already proceeded to total knee arthroplasty, and 1 patient had undergone secondary MACI at the same medial femoral condylar site because of an earlier graft failure. Therefore, 22 patients (10.8%) essentially had graft failure over the period. At the final follow-up, patients who underwent MACI in the tibiofemoral (vs patellofemoral) joint reported significantly better Knee injury and Osteoarthritis Outcome Score subscale scores for Quality of Life (*P* = .010) and Sport and Recreation (*P* < .001), as well as a greater knee extensor strength LSI (*P* = .002). Even though the tibiofemoral group demonstrated better 10-year MOCART scores for tissue infill (*P* = .027), there were no other MRI-based differences (*P* > .05).

**Conclusion:**

This study reports the long-term review of a prospective series of patients undergoing MACI, demonstrating good clinical scores, high levels of patient satisfaction, and acceptable graft survivorship at 10 years. Patients undergoing tibiofemoral (vs patellofemoral) MACI reported better long-term clinical outcomes, despite largely similar MRI-based outcomes.

The repair of symptomatic articular cartilage lesions remains a challenge given their limited potential for self-healing. Several surgical cartilage repair options are available, including bone marrow stimulation procedures such as microfracture,^31^ osteoarticular transplantation systems or mosaicplasty,^19^ and cell-based regeneration techniques such as autologous chondrocyte implantation (ACI).^5^ Traditional ACI procedures required the injection of cells suspended under a periosteal (first generation)^5^ or biodegradable collagen (second generation)^4^ membrane that was sutured to the adjacent cartilage surrounding the chondral defect. Third-generation matrix-induced ACI (MACI) seeds chondrocytes directly onto a type 1 or 3 collagen membrane, with the membrane subsequently fixed to subchondral bone at the base of the lesion using fibrin glue. Longer-term outcomes of techniques such as microfracture and osteoarticular transplantation system have been reported,^18,29,30,33^ and although these options may be better suited to smaller cartilage lesions, studies have reported inferior midterm outcomes for repair procedures such as microfracture when compared with third-generation MACI.^2,6,28^

However, despite the encouraging midterm outcomes of MACI,^3,6,11,12,16,35^ there are still only limited studies reporting longer-term (10 years) outcomes.^1,8,10,17,24^ Furthermore, most of these are in smaller patient cohorts and include mixed repair sites with no site-specific comparison of outcomes. Later-stage clinical and radiological patient follow-up, also with an investigation into the success of MACI based on tibiofemoral or patellofemoral graft location, is required to better assess clinical status and longer-term patient satisfaction, longevity of repair tissue, and provide improved education to patients for the setting of realistic short- and longer-term expectations.

The current prospective study sought to present the 10-year clinical and radiological outcomes in patients undergoing MACI and to compare clinical and magnetic resonance imaging (MRI)–based outcomes in patients undergoing tibiofemoral versus patellofemoral MACI. It was hypothesized that (1) a significant improvement in patient-reported outcome measures (PROMs) would be observed over the pre- and postoperative timeline to 10 years, although with no significant change from 2 to 10 years after surgery; (2) a high level of patient satisfaction (≥85% of patients) would be observed at the final postoperative follow-up; (3) no significant change in MRI-based outcomes would be observed from 2 to 10 years; and (4) no differences in clinical scores (PROMs and strength measures) or MRI-based outcomes would be observed between those undergoing tibiofemoral and patellofemoral MACI, specifically at 10 years after surgery.

## Methods

### Participants

**Figure 1.**
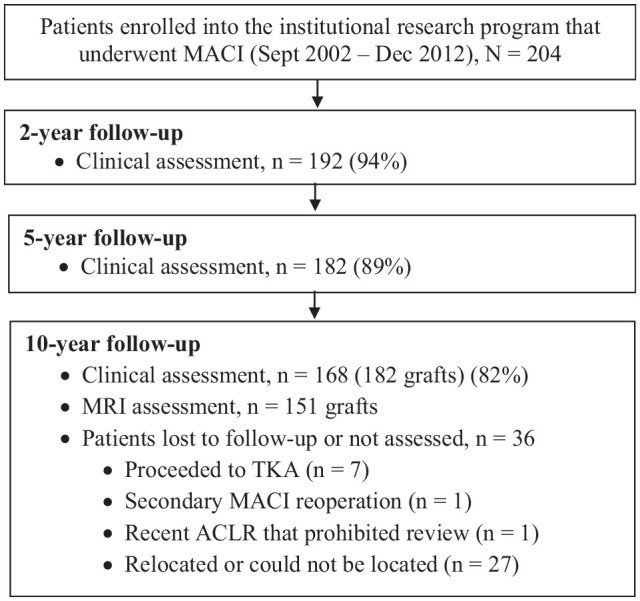
Flowchart demonstrating patients enrolled in the institutional research program who underwent matrix-induced autologous chondrocyte implantation (MACI), together with clinical and radiological evaluation of patients over the postoperative period. ACLR, anterior cruciate ligament reconstruction; MRI, magnetic resonance imaging; TKA, total knee arthroplasty.

Between September 2002 and December 2012, 204 patients were prospectively recruited into an institutional research program and underwent MACI. Although patients were assessed presurgery and at 2, 5, and 10 years (range, 10-16 years) after surgery, of the 204 patients initially recruited, 168 patients (182 grafts) were assessed at the final review, with 151 grafts undergoing MRI at the final follow-up (Figure 1). Table 1 reports descriptive characteristics and injury and surgery history for the cohort reviewed at 10 years.

**Table 1:** Preoperative Characteristics, Injury History, and Surgical Variables for the Cohort Assessed at 10 Years After Surgery, as Well as Those Who Underwent MACI in the Tibiofemoral or Patellofemoral Knee Joint^*a*^

Variable	Mean (range)	Tibiofemoral	Patellofemoral	*P*
Age, y	37.1 (16-58)	36.7 (16-58)	37.9 (18-58)	.810
Weight, kg	80.3 (46-130)	80.7 (46-130)	79.4 (56-117)	.645
Body mass index	26.1 (18.4-39.5)	26.3 (18.4-39.5)	25.5 (19.5-33.9)	.523
Defect size, cm^2^	3.2 (1.0-10.0)	3.2 (1.0-10.0)	3.3 (1.5-7.5)	.458
Previous procedures, n	1.2 (0-4)	1.2 (0-4)	1.3 (0-4)	.555
Duration of symptoms, y	8.1 (1-36)	8.3 (1-36)	7.5 (1-20)	.231
Defect location, (MFC/LFC/trochlea/patella), n	83/32/35/32	115	67	N/A

a Data are presented as mean (range) unless otherwise indicated. *P* values (independent *t* test) represent the tibiofemoral versus patellofemoral comparisons. LFC, lateral femoral condyle; MACI, matrix-induced autologous chondrocyte implantation; MFC, medial femoral condyle.

Indications for MACI surgery included being 15 to 65 years of age and verbally willing to adhere to a structured postoperative rehabilitation program. Preoperative MRI was undertaken in all patients to assess the location and size of the chondral defect, as well as concomitant pathology. Even though the indication for MACI was not dictated by the durations of symptoms or requirement to initially trial nonoperative management and/or other treatments, all patients had symptomatic, full-thickness grade 3 or 4 chondral lesions per the International Cartilage Regeneration & Joint Preservation Society classification system.^7^ Patients were not deemed candidates for MACI if they had ligamentous instability, had varus or valgus abnormalities (>3° tibiofemoral anatomic angle), had undergone previous extensive meniscectomy, or had ongoing progressive inflammatory arthritis. Patients with joint malalignment were included and underwent MACI if malignment was addressed at the time of surgery. Therefore, of the 168 patients with 10-year review, those with tibiofemoral malalignment (n = 4) underwent an offloading osteotomy if evaluated with significant varus or valgus lower limb deformity (as indicated by a >3° tibiofemoral anatomic angle), whereas those with patellofemoral malalignment (assessed via computed tomography imaging and >0.9-cm lateralization of tibial tuberosity) underwent Fulkerson osteotomy (n = 26). Furthermore, other concomitant surgeries performed specifically at the time of MACI included anterior cruciate ligament reconstruction (n = 6), posterior cruciate ligament reconstruction (n = 2), isolated lateral release (n = 8), and partial meniscectomy (n = 8). Although patients were appropriately consented for surgery, written informed consent was also attained before surgery and preoperative clinical review for research participation, per the institutional Human Research Ethics Committee approved by the Hollywood Private Hospital (HPH145).

### MACI Surgical Procedure and Rehabilitation

**Figure 2.**
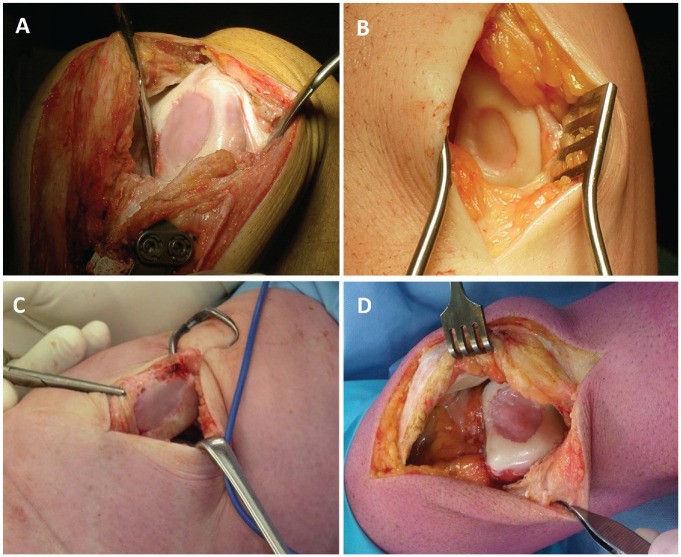
Intraoperative images demonstrating the final placement of a matrix-induced autologous chondrocyte implantation graft on the (A) medial femoral condyle, (B) lateral femoral condyle, (C) patella, and (D) trochlea.

The 2-stage MACI surgical procedure has been previously described.^8,13^ The procedure requires a sample of articular cartilage to be harvested arthroscopically, subsequent chondrocyte isolation and cell culturing (Genzyme), seeding of cells onto a type 1 or 3 collagen membrane (ACI-Maix Matricel GmbH), and reimplantation of the membrane during a second surgery via an open arthrotomy (Figure 2). All patients underwent a postoperative, graduated rehabilitation program, which has been previously described in detail.^10,13,15^ After the early inpatient hospital period, the program included a graduated increase in knee range of motion and weightbearing (with full weightbearing generally attained by 8-12 weeks), as well as progressive exercises prescribed to improve lower limb and trunk strengthening, functional weightbearing capacity, and activity- and sport-related tasks. The progression in these activities was further dictated by concomitant surgeries, graft location, and the individual patient’s conditioning and tolerance to exercise.

### Clinical Assessment

Patients were assessed presurgery and at 2, 5, and 10 years (range, 10-16 years) after surgery using a visual analog scale (VAS) for pain to assess the frequency and severity of knee pain on a scale of 0 to 10, as well as the Knee injury and Osteoarthritis Outcome Score (KOOS)^27^ to assess Pain, Symptoms, Activities of Daily Living (ADL), Sport and Recreation (Sport), and knee-related Quality of Life (QOL). At the final 10-year review, a patient satisfaction questionnaire was used to evaluate overall satisfaction, as well as satisfaction with the surgery to relieve pain, improve one’s ability to perform daily activities, and improve one’s ability to return to recreational activities and participate in sport. At 2, 5, and 10 years postoperatively, peak isokinetic knee extensor (quadriceps) and flexor (hamstring) strength was assessed at an angular velocity of 90 deg/s using an isokinetic dynamometer (Isosport International).

### Magnetic Resonance Imaging

High-resolution MRI using a Siemens Symphony 1.5 or 3 T-scanner (Siemens) was used postoperatively to assess graft repair per the MOCART (magnetic resonance observation of cartilage repair tissue) system.^22,26,32,34^ Individual parameters of graft status were assessed (scored as 1-4: 1 = poor, 2 = fair, 3 = good, 4 = excellent) in comparison with the adjacent native cartilage, including tissue infill, signal intensity, border integration, surface contour, tissue structure, effusion, subchondral lamina, and bone.^23^ An overall MRI graft composite score that incorporated these variables was also calculated.^9,26^ The MOCART scoring tool is shown in Table 2.

**Table 2:** Pertinent Parameters of Graft Repair, as Well as Weighting Scales Employed to Calculate the MRI Composite Score, for the MOCART System^*a*^

Parameter	Score	Description	Weighting
Signal intensity	1 = poor	Fluid signal/hyperintense diffuse	0.3
	2 = fair	Hyperintense basal layer >50%/<50%	
	3 = good	Hypointense	
	4 = excellent	Isointense	
Graft infill	1 = poor	Subchondral bone exposed	0.2
	2 = fair	<50% height of adjacent cartilage	
	3 = good	>50% height of adjacent cartilage	
	3.5 = very good	Hypertrophy	
	4 = excellent	Complete infill	
Border integration	1 = poor	Incomplete border, visible defect	0.15
	2 = fair	Incomplete border, split visible	
	3 = good	Complete border, minor split	
	4 = excellent	Complete integration	
Surface contour	1 = poor	Ulceration, delamination, full thickness	0.1
	2 = fair	<50% surface fibrillation	
	3 = good	Focal changes only	
	4 = excellent	Smooth surface	
Structure	1 = poor	Heterogeneous, clefts	0.1
	2 = fair	Heterogeneous, no clefts	
	3 = good	>50% homogeneous	
	4 = excellent	>75% homogeneous	
Subchondral lamina	1 = poor	No visible lamina	0.05
	2 = fair	<25% intact	
	3 = good	>50% intact	
	4 = excellent	Fully reconstituted	
Subchondral bone	1 = poor	Cysts, sclerosis, edema	0.05
	2 = fair	Edema >1 cm from lamina	
	3 = good	Edema <1 cm from lamina	
	4 = excellent	Intact, no significant edema	
Effusion	1 = poor	Severe	0.05
	2 = fair	Moderate	
	3 = good	Mild	
	4 = excellent	None	

a MOCART, magnetic resonance observation of cartilage repair tissue; MRI, magnetic resonance imaging.

### Statistical Analysis

The mean, standard deviation, and range of all clinical and radiological measures were calculated and are presented. Peak isokinetic knee extensor and flexor strength measures are presented as limb symmetry indices (LSIs; the operated limb expressed as a percentage of the nonoperated limb). Subsequently, independent *t* tests were undertaken to compare pertinent preoperative patient and surgical characteristics (age, body mass index, defect size, and duration of symptoms), as well as 10-year clinical scores (KOOS subscales and isokinetic knee extensor LSIs) and MRI-based outcomes (graft infill and the MRI composite score), in patients with (1) tibiofemoral grafts undergoing concomitant offloading osteotomy (n = 4) versus not and (2) patellofemoral grafts undergoing concomitant Fulkerson osteotomy (n = 26) or not. No statistical differences (*P* > .05) were observed in any of the aforementioned variables; hence, patients were grouped and analyzed within the tibiofemoral and patellofemoral groups irrespective of whether they underwent concomitant osteotomy or not. To evaluate changes in clinical scores over time, we used analysis of variance. The cohort was further stratified based on defect location, including those undergoing tibiofemoral (medial femoral condyle [MFC] and lateral femoral condyle) and patellofemoral (patellar and trochlear) MACI with 10-year follow-up. Group comparisons were then made using independent *t* tests for preoperative patient characteristics, injury and surgery variables, and 10-year clinical and MRI-based measures. Statistical analysis was performed using SPSS software (Version 27.0; IBM Corp), with statistical significance determined at *P* < .05.

## Results

### Clinical Review

All PROMs significantly improved over the pre- to postoperative period to 2 years after surgery (Table 3). The peak knee extensor strength LSI significantly increased from 2 to 10 years (Table 3), with no changes in the peak knee flexor strength LSI (Table 3). At the final follow-up, 92% of patients were satisfied with the knee pain relief provided by MACI, 76% were satisfied with their ability to participate in sports, and 89% were satisfied overall (Table 4).

**Table 3:** Patient-Reported Outcome Measures and Knee Extensor and Flexor Strength LSIs Throughout the Pre- and Postoperative Timeline^*a*^

Score	Preoperative	Postoperative			*P*
		2 y	5 y	10 y	
KOOS Pain	63.7 (18.2) [11.1-100]	84.3 (12.8) [47.2-100]	85.5 (13.5) [33.3-100]	85.0 (13.9) [27.8-100]	<.0001
KOOS Symptoms	66.1 (18.5) [3.6-100]	86.1 (11.3) [50.0-100]	85.0 (13.2) [21.4-100]	84.2 (14.8) [21.4-100]	<.0001
KOOS ADL	73.6 (18.1) [20.6-100]	90.3 (11.7) [35.3-100]	90.6 (11.5) [36.8-100]	90.3 (13.1) [35.3-100]	<.0001
KOOS Sport	26.3 (23.5) [0-100]	58.1 (29.6) [0-100]	61.9 (29.8) [0-100]	66.6 (26.7) [0-100]	<.0001
KOOS QOL	28.6 (19.3) [0-100]	58.2 (22.9) [0-100]	60.5 (25.3) [0-100]	63.2 (23.1) [0-100]	<.0001
VAS-F	5.9 (2.4) [0-10]	2.3 (2.3) [0-10]	2.3 (2.4) [0-10]	2.2 (2.3) [0-10]	<.0001
VAS-S	4.3 (2.3) [0-10]	2.0 (1.5) [0-7]	2.0 (1.6) [0-7]	1.9 (1.5) [0-7]	<.0001
Knee extensor strength LSI	NA	86.7 (20.4) [30.6-156.6]	89.4 (17.1) [34.7-142.5]	93.8 (16.2) [34.7-133.9]	.004
Knee flexor strength LSI	NA	101.1 (17.5) [66.9-167.9]	100.0 (18.0) [51.8-156.8]	99.1 (13.1) [62.3-167.9]	.398

a Data are presented as mean (SD) [range]. *P* values represent change over time (analysis of variance), ADL, Activities of Daily Living; KOOS, Knee injury and Osteoarthritis Outcome Score; LSI, limb symmetry index; NA, not applicable; QOL, Quality of Life; Sport, Sport and Recreation; VAS-F, visual analog scale, frequency of pain; VAS-S, visual analog scale, severity of pain.

**Table 4:** Number of Patients Within Each of the 4 Satisfaction Gradings, for Each Satisfaction Item, for the Entire Cohort Reviewed at 10 Years After Surgery^*a*^

Satisfaction Item	Pain Relief	Improving Ability to Undertake ADL	Improving Ability to Participate in Recreational Activities	Improving Ability to Participate in Sport	Overall Satisfaction
Very satisfied	98	99	90	48	95
Satisfied	56	56	53	79	54
Dissatisfied	12	11	16	22	15
Very dissatisfied	2	2	9	19	4
Overall Satisfied	154 (91.7)	155 (92.3)	143 (85.1)	127 (75.6)	149 (88.7)

a Data are presented as n or n (%). ADL, Activities of Daily Living.

### MRI-Based Review

**Figure 3.**
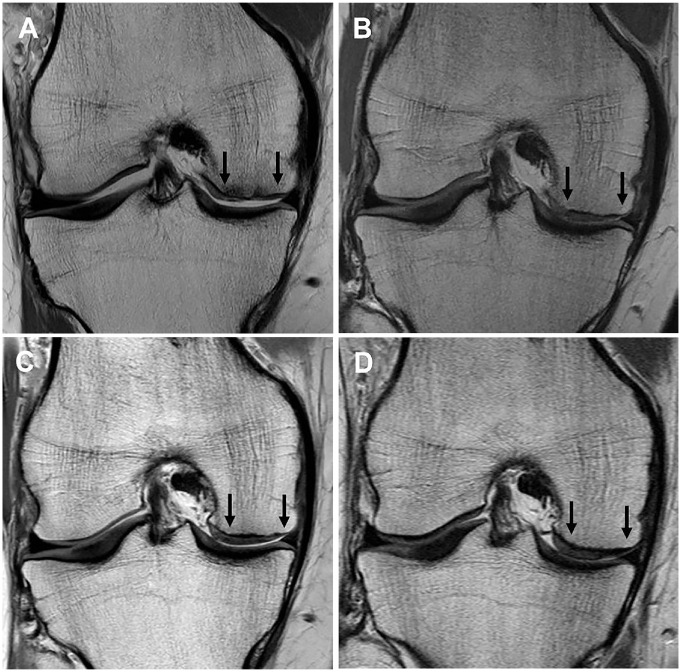
Proton density fast spin-echo magnetic resonance imaging scans of (A) a chondral defect preoperatively on the medial femoral condyle (between black arrows), as well as the subsequent matrix-induced autologous chondrocyte implantation graft at (B) 2 years, (C) 5 years, and (D) 10 years.

No significant change was observed for any MRI-based scoring variable, or the overall MRI composite score, for the full cohort from 2 to 10 years after surgery (Table 5). Of the 151 grafts reviewed via MRI scans at the final 10-year review, 55 grafts (36.4%) demonstrated excellent graft infill, 49 (32.5%) demonstrated good infill, 15 (9.9%) demonstrated fair infill, 14 (9.3%) demonstrated poor infill, and 18 grafts (11.9%) demonstrated an element of graft hypertrophy, per the MOCART scoring tool (Table 2). Images of a preoperative chondral defect on the MFC and the subsequent MACI graft at 2, 5, and 10 years after surgery are shown in Figure 3.

**Table 5:** Postoperative MRI Review of MACI Grafts^*a*^

Variable	Postoperative Time Point			*P*
	2 y	5 y	10 y	
Graft infill	3.3 (0.8) [1-4]	3.2 (0.8) [1-4]	3.1 (0.8) [1-4]	.200
Signal intensity	3.0 (0.70) [1-4]	2.9 (0.8) [1-4]	2.9 (0.7) [1-4]	.451
Border integration	3.1 (1.0) [1-4]	3.0 (1.0) [1-4]	2.9 (1.0) [1-4]	.189
Surface contour	3.1 (1.0) [1-4]	2.9 (1.0) [1-4]	2.8 (1.1) [1-4]	.104
Structure	3.3 (0.9) [1-4]	3.1 (0.9) [1-4]	3.0 (1.0) [1-4]	.099
Subchondral lamina	3.7 (0.5) [2-4]	3.6 (0.6) [2-4]	3.5 (0.6) [2-4]	.231
Subchondral bone	2.9 (1.1) [1-4]	2.9 (1.0) [1-4]	2.9 (1.0) [1-4]	.601
Effusion	3.7 (0.5) [2-4]	3.7 (0.5) [2-4]	3.6 (0.5) [2-4]	.512
MRI composite score	3.2 (0.6) [1.2-3.9]	3.1 (0.6) [1.2-4.00]	3.0 (0.6) [1.2-3.9]	.179

a Data are presented as mean (SD) [range]. *P* values represent changes over time within the full cohort (analysis of variance). MACI, matrix-induced autologous chondrocyte implantation; MRI, magnetic resonance imaging.

### Graft Failures

Of the 151 grafts reviewed via MRI scans at the final 10-year review, 14 (9.3%) had failed (defined by graft delamination or no discernible graft tissue on MRI scans). Furthermore, of the 36 patients (of the prospectively recruited 204) who were not available for longer-term review, 7 had already proceeded to total knee arthroplasty (TKA), and 1 patient had undergone secondary MACI at the same MFC site because of earlier graft failure. Therefore, 22 patients (10.8%) essentially had graft failure at or before the final review time.

### Tibiofemoral Versus Patellofemoral MACI Comparison

**Figure 4.**
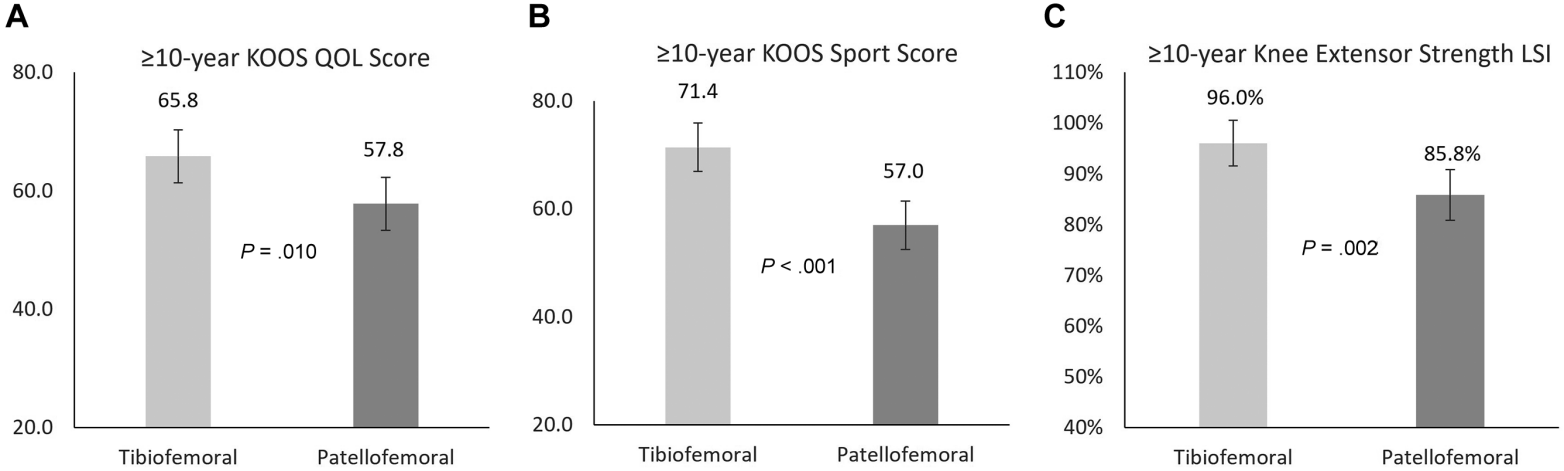
Comparison of the (A) Knee injury and Osteoarthritis Outcome Score (KOOS) Quality of Life (QOL) subscale, (B) KOOS Sport and Recreation (Sport) subscale, and (C) peak knee extensor strength limb symmetry index (LSI) between patients undergoing tibiofemoral and patellofemoral matrix-induced autologous chondrocyte implantation at 10-year postoperative review.

No group differences were observed between patients who underwent tibiofemoral and patellofemoral MACI in preoperative descriptive and injury or surgery variables (Table 1) or between preoperative PROMs (Appendix Table A1, available in the online version of this article). At the final 10-year follow-up, patients who underwent MACI in the tibiofemoral (versus patellofemoral) joint demonstrated a significantly better MOCART tissue infill score (*P* = .027; tibiofemoral mean, 3.2; patellofemoral mean, 2.9) (Table 6), although there were no other differences in MRI-based scores, including the overall MRI composite score (*P* = .481; tibiofemoral mean, 3.0; patellofemoral mean, 3.1) (Table 6). Patients undergoing tibiofemoral (vs patellofemoral) MACI reported significantly better 10-year KOOS subscale scores for QOL (*P* = .010; tibiofemoral mean, 65.8; patellofemoral mean, 57.8) (Figure 4A) and Sport (*P* < .001; tibiofemoral mean, 71.4; patellofemoral mean, 57.0) (Figure 4B), as well as a greater knee extensor strength LSI (*P* = .002; tibiofemoral mean, 96.0%; patellofemoral mean, 85.8%) (Figure 4C).

**Table 6:** Statistical Comparison of Graft Infill and the MRI Composite Score at 10 Years Between Those Undergoing MACI in the Tibiofemoral or Patellofemoral Knee Joint^*a*^

Graft Location	Graft Size	Graft Infill Score	MRI Composite Score
MFC	3.4 (2.2) [1.0-10.0]	3.2 (0.8) [1.0-4.0]	3.0 (0.6) [1.2-4.0]
LFC	2.7 (1.4) [1.0-7.5]	3.1 (0.8) [1.0-4.0]	2.9 (0.6) [1.4-3.8]
Trochlea	3.5 (1.8) [1.0-7.5]	2.8 (0.8) [1.0-4.0]	3.0 (0.6) [1.2-3.6]
Patella	3.1 (1.3) [1.4-6.0]	3.0 (0.9) [1.0-4.0]	3.3 (0.6) [1.2-3.8]
Tibiofemoral	3.2 (2.0) [1.0-10.0]	3.2 (0.8) [1.0-4.0]	3.0 (0.6) [1.2-4.0]
Patellofemoral	3.3 (1.5) [1.0-7.5]	2.9 (0.9) [1.0-4.0]	3.1 (0.6) [1.2-3.8]
*P* value (tibiofemoral vs patellofemoral)	.458	.027	.481

a Data are presented as mean (SD) [range]. LFC, lateral femoral condyle; MACI, matrix-induced autologous chondrocyte implantation; MFC, medial femoral condyle; MRI, magnetic resonance imaging.

## Discussion

This prospective study reports the 10-year clinical and MRI-based outcomes in patients undergoing MACI. These findings demonstrate improved clinical scores, high levels of patient satisfaction, clinical and MRI-based outcomes that were largely sustained during the 2 to 10 years after surgery, and an acceptable graft failure rate over the assessment period. Furthermore, patients undergoing tibiofemoral (vs patellofemoral) MACI reported better 10-year clinical outcomes (KOOS QOL and Sport subscales, as well as knee extensor strength symmetry), as well as degree of tissue infill, despite a similar overall MRI composite score.

As expected, PROMs significantly improved as a result of surgery, with the maximal improvement at 2 years and no significant change in any PROM from 2 years to the final review at 10 years after surgery. This was in support of the first hypothesis and reinforces the sustained longer-term clinical benefit offered by MACI as a cartilage regeneration procedure for symptomatic cartilage lesions. It should be acknowledged that only 82% of the recruited cohort was available for 10-year review, with 75% of the nonresponders either having relocated or being unable to be located. It is unknown how this cohort may have potentially skewed the 10-year results, although none of these patients had returned for orthopaedic consultation seeking further treatment. Furthermore, even though an additional 7 patients who had already progressed to TKA were included in the overall graft failure rate reported, they could not be assessed at 10 years. Therefore, although it may be unlikely that this small cohort would significantly skew (reduce) the mean PROMs reported should they have delayed their TKA beyond 10 years and been available for 10-year clinical review, their 10-year PROMs could not be included.

Nonetheless, although improved PROMs have been reported in the limited studies reporting longer-term outcomes after MACI,^1,8,10,17,24^ the 10-year KOOS subscale outcomes in the current study were still similar to or better than those reported previously using the KOOS.^1,8,10^ Even though not used in the current study and making comparison of outcomes between some studies difficult, other studies reporting longer-term outcomes after third-generation MACI have demonstrated improvement in the International Knee Documentation Committee, Lysholm, Tegner, Noyes, and 36-item Short Form Health Survey scores.^1,8,17,24^ Furthermore, whereas only 76% of patients were satisfied with their 10-year ability to participate in sports, in support of the second hypothesis, >85% of patients were satisfied with their outcome overall, as well as with pain relief and their ability to undertake ADL and participate in recreational activities. These satisfaction rates are similar to those previously reported.^8,10^

Although we currently lack longer-term MRI-based outcomes after third-generation MACI, it was encouraging in this larger cohort that no significant change (deterioration) was observed in MRI-based parameters of graft repair, which would suggest an appropriate degree of repair tissue sustainability and supports the third hypothesis. Of interest, a more recently published case series reported significant deterioration in MRI-based tissue structure and subchondral lamina MOCART parameters between 2 and 10 years, despite no significant change in tissue infill or overall MRI graft composite score.^10^ Of the limited longer-term studies reporting MRI-based outcomes after third-generation MACI, sound repair tissue survivorship has been demonstrated.^1,8,10,24^

The fourth hypothesis was not supported when we investigated 10-year clinical and MRI-based outcomes between those undergoing tibiofemoral and patellofemoral MACI. Despite no significant differences in the VAS pain scores or the majority of KOOS subscales (Pain, Symptoms, and ADL), those undergoing tibiofemoral (vs patellofemoral) MACI reported significantly better longer-term scores for the KOOS subscales of QOL and Sport. Better KOOS QOL and Sport subscale scores have been previously reported in the short-term (2 years) follow-up of tibiofemoral versus patellofemoral MACI,^14^ although that study did not report any strength-based outcomes. Albeit not previously assessed after MACI, an association between quadriceps strength asymmetry and decreased knee-related function has been reported in patients after anterior cruciate ligament reconstruction.^21^ Although unsubstantiated, the reduced quadriceps strength LSIs observed in the patellofemoral cohort in the current study may be linked with the reporting of those specific KOOS subscale PROMs, and the result of the increased trauma to the extensor mechanism at the time of surgery and failure to restore lingering quadriceps strength deficits after surgery. Of interest, whereas the patellofemoral (vs tibiofemoral) cohort demonstrated a significantly lower degree of graft tissue infill at 10 years, no other MRI-based variables (including the overall MRI composite score) demonstrated group-based differences.

Some limitations should be acknowledged in the current study. First, despite a robust postoperative clinical and radiological follow-up to 10 years, no comparative cohort was investigated. As previously reported, other cartilage repair surgical procedures have been reported^19,31^ and may be deemed suitable, particularly for smaller chondral lesions (≤4 cm^2^), although within our geographical location MACI was considered the standard technique and routinely used throughout the designated recruitment period. Second, whereas the current study sought to assess the KOOS and VAS score, a range of other PROMs (including specific activity-based PROMs) have been used and reported in the evaluation of patients who underwent MACI and may provide further insight into the physical recovery profile of patients. Furthermore, in the absence of any differences in the KOOS Pain, Symptoms, and ADL subscales, we do not know the underlying reasons (outside of variables such as greater quadriceps strength asymmetry) for the patellofemoral versus tibiofemoral group–based differences in KOOS QOL and Sport subscales. As mentioned previously, it is unknown how the inability to include clinical scores from nonresponders at 10 years may have skewed results. Furthermore, although the patients who had already undergone TKA before their 10-year postoperative review were included in the overall graft failure rate provided, they were not available to contribute 10-year PROMs. Therefore, we acknowledge that inclusion of their data if they had delayed their TKA beyond 10 years may have reduced the mean PROMs reported.

Although a significant difference in quadriceps strength LSIs was observed postoperatively between patients who underwent tibiofemoral and patellofemoral MACI, strength was not assessed preoperatively (and realistically would have been affected regardless by underlying pain and symptoms that drove patients toward cartilage repair surgery intervention). We are therefore unable to ascertain how much limb strength asymmetry was also present preoperatively (especially given the mean preoperative duration of symptoms reported by patients) or whether the primary contribution came specifically as a result of the surgery and the subsequent inability to restore deficits as a result of the rehabilitation intervention. Finally, the current study sought to investigate the 10-year MRI-based outcome of the MACI graft rather than any widespread changes throughout the knee. Although plain film radiographs were not undertaken postoperatively, future studies may further seek to utilize MRI-based tools to evaluate whole-organ osteoarthritic changes in the knee, such as the Whole-Organ Magnetic Resonance Imaging Score^25^ or MRI Osteoarthritis Knee Score,^20^ with respect to tibiofemoral and patellofemoral MACI and separate from the assessment of graft status in isolation.

## Conclusion

This prospective study demonstrates improved clinical scores, high levels of patient satisfaction, clinical and MRI-based outcomes that were largely sustained during the 2 to 10 years after surgery, and an acceptable graft failure rate over the assessment period. Furthermore, despite MRI-based scores that were largely similar based on graft location (apart from the degree of repair tissue infill), patients undergoing tibiofemoral (vs patellofemoral) MACI reported better 10-year clinical outcomes and knee extensor strength symmetry. Longer-term outcomes after third-generation MACI are lacking, with the current study providing information to the surgeon and therapist to provide more accurate patient education for the setting of realistic short- and longer-term expectations when considering MACI.

## Supplemental Material

sj-pdf-1-ajs-10.1177_03635465241227969 – Supplemental material for 10-Year Prospective Clinical and Radiological Evaluation After Matrix-Induced Autologous Chondrocyte Implantation and Comparison of Tibiofemoral and Patellofemoral Graft Outcomes Supplemental material, sj-pdf-1-ajs-10.1177_03635465241227969 for 10-Year Prospective Clinical and Radiological Evaluation After Matrix-Induced Autologous Chondrocyte Implantation and Comparison of Tibiofemoral and Patellofemoral Graft Outcomes by Jay R. Ebert, Minghao Zheng, Michael Fallon, David J. Wood and Gregory C. Janes in The American Journal of Sports Medicine
