# Supplementary material for: 10-Year Prospective Clinical and Radiological Evaluation After Matrix-Induced Autologous Chondrocyte Implantation and Comparison of Tibiofemoral and Patellofemoral Graft Outcomes
Source: Am J Sports Med. 2024 Feb 21;52(4):977–86. doi: 10.1177/03635465241227969 (PMC10943616; doi:10.1177/03635465241227969)
Supplement: sj-pdf-1-ajs-10.1177_03635465241227969 – Supplemental material for 10-Year Prospective Clinical and Radiological Evaluation After Matrix-Induced Autologous Chondrocyte Implantation and Comparison of Tibiofemoral and Patellofemoral Graft Outcomes [file sj-pdf-1-ajs-10.1177_03635465241227969.pdf]

Appendix

Table A1. Patient-reported outcome measures (PROMs) reported by both the tibiofemoral and patellofemoral MACI cohorts throughout the pre- and post-operative timeline. Shown are means (SD).

Time-point	Group	KOOS (Pain)	KOOS (Symptoms)	KOOS (ADL)	KOOS (Sport)	KOOS (QOL)	VAS-F	VAS-S
Pre-surgery	Tibiofemoral	65.8 (19.4)	67.7 (18.0)	76.8 (18.1)	26.2 (25.7)	29.9 (22.3)	5.8 (2.8)	4.2 (2.3)
	Patellofemoral	63.4 (15.6)	65.2 (17.2)	73.1.0 (16.1)	24.6 (21.0)	27.9 (15.9)	6.2 (1.6)	4.4 (0.9)
2 years	Tibiofemoral	84.7 (13.3)	86.0 (11.8)	91.4 (11.8)	61.2 (29.2)	60.0 (22.6)	2.4 (2.5)	2.0 (1.7)
	Patellofemoral	83.3 (11.4)	86.4 (9.8)	87.5 (11.0)	50.1 (29.4)	53.3 (23.0)	2.1 (1.4)	1.9 (1.1)
5 years	Tibiofemoral	87.4 (13.1)	85.3 (14.6)	93.1 (10.0)	67.6 (26.6)	62.6 (24.6)	2.5 (2.7)	2.1 (1.7)
	Patellofemoral	83.3 (11.4)	84.8 (9.8)	87.5 (11.0)	50.1 (29.4)	53.3 (23.0)	2.1 (1.4)	1.8 (1.1)
10 years	Tibiofemoral	85.5 (15.2)	83.2 (16.4)	91.5 (13.7)	71.4 (29.2)	65.8 (25.2)	2.2 (2.5)	1.9 (1.7)
	Patellofemoral	84.0 (10.1)	85.4 (9.8)	87.5 (11.0)	57.0 (18.3)	57.8 (16.9)	2.1 (1.4)	1.8 (1.1)

KOOS = Knee Injury and Osteoarthritis Outcome Score; ADLs = Activities of Daily Living; QOL = Quality of Life; VAS-F = Visual Analogue Pain Scale (Frequency of Pain); VAS-S = Visual Analogue Pain Scale (Severity of Pain).
